# Salmon Aquaculture and Transmission of the Fish Tapeworm

**DOI:** 10.3201/eid1301.060875

**Published:** 2007-01

**Authors:** Felipe C. Cabello

**Affiliations:** * New York Medical College, Valhalla, New York, USA

**Keywords:** diphyllobothriasis, aquaculture, cestode infections, food, commentary

Aquaculture of salmon constitutes a rapidly growing worldwide industry with an expanding globalized market (*1**,**2*). Although this industry has several economic benefits, according to recent reports it is also accompanied by effects that are detrimental to human and animal health and the environment (*1**,**2*). Aquaculture has been implicated in the transmission of infectious diseases. For example, in caged fish aquaculture, bacterial and parasitic diseases can be transmitted to wild fish (*1**,**2*). Furthermore, aquaculture-raised fish may be susceptible to the microorganisms and parasites of wild fish (*1**,**3*). However, in spite of the accepted fact that parasitic worms can be transmitted to humans by free-ranging fish (4), until recently, few examples have been reported of pathogens that could be transmitted to humans directly by the products and subproducts of salmon aquaculture. I discuss here information indicating that salmon aquaculture is involved in expanding the range of fish tapeworm infections in nature and to humans.

Several recent publications report outbreaks of human cases of infection by the fish tapeworm Diphyllobothrium latum in Brazil (*5**–**9*). These infections have been epidemiologically linked to consumption of raw salmon produced by the aquaculture industry in southern Chile, thousands of miles away (*5**–**9*). Infections by D. latum have been detected in several cities in Brazil (*5**–**9*), and in 1 tourist who traveled there from Europe (*10*). These cases of diphyllobothriasis are noteworthy because this parasite was totally unknown to clinicians and parasitologists in Brazil, where it does not appear to have an endemic life cycle (*5**–**9*).

D. latum is transmitted to humans by plerocercoid larvae present in fish meat and visceral organs (http://www.dpd.cdc.gov/dpdx). D. latum and the closely related sea gull tapeworm, D. dendriticum, have well-established endemic life cycles in a series of glacial lakes that dot Region XIX and Region X in northern Chilean Patagonia. Infections with these parasites have been detected in this geographic area since the 1950s in persons who ingested uncooked fish from these lakes and also in animals (*11**–**14*). The link that closes the epidemiologic chain between the Brazilian outbreak of fish tapeworm infections and the aquaculture of salmon in southern Chile is that some of the freshwater lakes where D. latum and D. dendriticum are endemic are used to grow the freshwater stages of juvenile salmon, or smolt, in cages (*15*). Smolt are temporarily grown in these lakes to accelerate their growth before they are transported to cages in the sea where the salmon will reach adult stages. The practice of growing smolt in freshwater lakes appears to be unique to Chilean salmon aquaculture; in other salmon aquaculture settings, smolt are grown in tanks containing filtered water.

During the past 55 years, work by Chilean parasitologists has demonstrated that native species and introduced salmonid fish are infested with Diphyllobothrium plerocercoids in these lakes (*11**–**14*). Moreover, the other intermediary hosts of the fish tapeworm, the calanoid copepods Diaptomus diabolicus and Boeckela gracilipes, are also abundant (*16*). Native and introduced fish ingest copepods containing procercoid larvae that develop into plerocercoids (*16*). The fish tapeworm life cycle is subsequently closed in these lakes when humans and animals, the definitive hosts of these fish tapeworms, ingest infested fish (*11**–**16*). The persistence of the cycle of D. latum in these lakes is facilitated by the release of untreated sewage, which deposits stools of infected humans containing high concentrations of fish tapeworm eggs in the water (*11**–**17*).

The Brazilian studies did not detect Diphyllobothrium plerocercoids in several samples of Chilean salmon tested after the first human cases of diphylobothriasis appeared (*7*). However, this failure may have resulted from limited sampling or temporal and spatial variability in the infestation of the salmon with plerocercoids (*11**–**17*). Nonetheless, recent work in Chile has demonstrated the presence of Diphyllobothrium plerocercoids in rainbow trout raised in aquaculture, which suggests that aquacultured fish can become infected with these parasites (*18*). In Chile, infestation with Diphyllobothrium plerocercoids has also been detected in coho salmon living in the wild, a nonindigenous species raised originally in aquaculture that escaped from pens (*19*). Larvae of another fish tapeworm, D. pacificum, whose definitive hosts are large marine mammals such as sea lions and fur seals, have been detected in marine fish in Chile (*20*). Salmon aquaculture sea cages attracts these large mammals, creating the possibility for the parasite life cycle to occur in the environment around the salmon cages (*20*). However, this is an unlikely scenario for the spread to human populations, because the fish tapeworms identified in the patients in Brazil had the morphologic characteristics of D. latum, which as discussed above is one of the diphyllobothrium endemic in the lakes of southern Chile (*5**–**9*). These findings suggest that the aquaculture of salmon in southern Chile has expanded the species range of infestation by diphyllobothrium to nonindigenous salmonid fish species introduced by the aquaculture industry (*18**,**19*) and that the escape of infected fish from aquaculture sea cages has probably resulted in the expansion of the geographic range of the disease in Chile (*19*). In turn, the marketing of Chilean aquacultured salmon in Brazil has expanded the range of this human disease to a geographic region where this pathology was until now absent (*5**–**9*).

Traditionally in Europe and North America, infections with fish tapeworms were incurred during the preparation of gefilte fish by Jewish women who tasted bits of uncooked freshwater fish and thus ingested plerocercoids (*21*). In Chile, infestation of humans with the fish tapeworm in the D. latum–endemic area results from ingestion of raw and smoked fish, and in the Brazilian outbreak all the case-patients had previously eaten salmon sushi. Marinated ceviches may also be able to transmit infecting plerocercoids (*5**–**9**,**11**–**17*). The disease in humans can be prevented by cooking the fish at a temperature of 54°C to 56°C for 5 minutes (*21*). Alternatively, the plerocercoids can be destroyed by blast-freezing the fish at –35°C for 15 hours and by regular freezing at –20°C for 7 days before consumption (*22*).

Thus, to avoid new human outbreaks of fish tapeworm in other geographic areas where this parasite does not exist, salmon originated from aquaculture should not be eaten raw, at least not until it has been frozen under the conditions discussed above. Assuming the epidemiologic information presented here explains the appearance of the fish tapeworm outbreak in Brazil, it would be preferable, in terms of sanitation, for the Chilean aquaculture industry to stop growing salmon smolt in the lakes in the areas where diphyllobothriasis is endemic in humans and animals (*11**–**17*).

This epidemiologic event may also be understood as cautionary tale, and 1 more example of the dangers entailed by the globalization of food supply and of the rapidly changing global eating habits that facilitate the distribution of human and animal pathogens worldwide. The expansion of diphyllobothriasis-endemic areas in Chile may, in turn, facilitate the appearance of future outbreaks of this disease as the aquaculture industry expands to these new infested areas and the market for Chilean salmon enlarges worldwide. The increased popularity of eating uncooked fish in sushi and ceviche will also be a factor in the emergence of future outbreaks of this disease (*4**,**21*). As has been the case with other human infectious diseases disseminated by the industrialization of animal husbandry, this outbreak of diphyllobothriasis could have been prevented by use of existing information, including that concerning the endemic nature of diphyllobothriasis in the lakes of southern Chile and its transmission by raw fish.
